# 

**Published:** 1872-01

**Authors:** 


### CONTENTS:

RUSH MEDICAL COLLEGE — Twenty-ninth Annual Commencement Exercises:

Charge to the Class. By Dr. J. W. Freer .... 3

Class Valedictory. By O. J. H. Adams, 5

Valedictory. By Prof. Jas. H. Etheridge .... 10

MILITARY TRACT MED. ASSOCIATION—Thirteenth Semi-Annual Meeting .... 22

ANÆSTHESIA, HYPERÆSTHESIA — Introductory Lecture by Walter Hay, M.D. 28

EDITORS' BOOK TABLE .... 40

EDITORIAL .... 43

LOOT .... 45

